# Uncemented cups with and without screw holes in primary THA: a Swedish Hip Arthroplasty Register study with 22,725 hips

**DOI:** 10.1080/17453674.2019.1599777

**Published:** 2019-04-08

**Authors:** Volker Otten, Sebastian Mukka, Kjell Nilsson, Sead Crnalic, Johan Kärrholm

**Affiliations:** a Department of Surgical and Perioperative Sciences (Orthopaedics), Umeå University, Umeå;; b Department of Orthopaedics, Institute of Surgical Science, Sahlgrenska University Hospital, Gothenburg University, Mölndal, Sweden

## Abstract

Background and purpose — Uncemented cups in total hip arthroplasty (THA) are often augmented with additional screws to enhance their primary stability. We investigated whether there is a difference in the risk for revision between cups with screw holes and cups without screw holes.

Patients and methods — We analyzed the risk for cup revision of uncemented cups registered in the Swedish Hip Arthroplasty Register (SHAR) between 2000 and 2017 with respe ct to the presence of screw holes. Only patients with primary osteoarthritis (OA) were included. 22,725 cups, including 12,354 without screw holes and 10,371 with screw holes, were evaluated. Revision rates at 2 and 10 years after the primary operation were analyzed.

Results — At a median follow-up time of 3.4 years (0–18), 459 cup revisions were reported. The main reasons for cup revision during the whole observation time were infection, 52% of all cup revisions, and dislocation, 26% of all cup revisions. The survival rate with cup revision due to aseptic loosening as endpoint was 99.9% (95% CI 99.8–99.9) at 2 years for both cups with and cups without screw holes, and the survival rates at 10 years were 99.5% (CI 99.3–99.7) and 99.1% (CI 98.6–99.5), respectively. Cups without screw holes showed a decreased risk of revision due to any reason at both 2 years (adjusted hazard ratio [HR] 0.6, CI 0.5–0.8) and 10 years (HR 0.7, CI 0.5–0.9).

Interpretation — We found a very low revision rate for aseptic loosening with modern, uncemented cup designs. Cups with screw holes had an increased risk of revision due to any reason in patients with primary OA

Uncemented cups have gained popularity in recent years, even in countries traditionally using cemented fixation (Kärrholm et al. 2017). Initial stability is essential for good long-term results. Therefore, uncemented cups are often augmented with screws or pegs. However, screw holes in acetabular cups have also been discussed as potential routes for synovial fluid, which might lead to osteolysis (Aspenberg and van der Vis 1998, Iorio et al. 2010). Additionally, using screws for cup fixation increases the cost and operation time. Hence, the surface of uncemented cups has been further developed towards a rougher finish to improve primary stability and long-term bone ingrowth even without any extra augmentation.

In a recently published randomized controlled study with a minimum follow-up of 14 years comparing cups with and without screw holes, we found no difference in implant migration (Otten et al. 2016). To our knowledge, there has not been any published study investigating the risk for cup revision depending on the use of cups with or without screws.

We analyzed the survival of uncemented cups with or without screw holes in the Swedish Hip Arthroplasty Register (SHAR). Our hypothesis was that screw fixation reduces the risk for early aseptic loosening but increases the risk for late failure because of potentially increased risk for osteolysis due to screw holes (Iorio et al. 2010). Revision rates due to aseptic loosening and for any reason at 2 and 10 years after primary operation were analyzed.

## Patients and methods

### Study design, source of data, and terminology

This study is based on data obtained from the SHAR. Primary and revision hip arthroplasties performed in Sweden have been registered since 1979. During the last decade, the completeness of primary surgeries has been approximately 98–99% and 94% for revisions. Since 1999, detailed information regarding the prosthesis design, such as the presence of screw holes, has been registered in the SHAR.

The guidelines of the STROBE statement were followed.

In this study, we classified cups without any holes and cups with 1 central hole, used for the cup impactor, into the category referred to as “cups without screw holes.” Cups with holes placed in a sector of the shell or over the whole cup area (multi-hole cups) were classified into “cups with screw holes” regardless of whether the holes were intended for screws or pegs, as long as the holes penetrated the entire thickness of the shell. Cup revision is defined as any reoperation of the hip where 1 or more components of the cup (shell, liner, or both) were removed or exchanged. “Aseptic loosening” as the reason for revision includes aseptic loosening, osteolysis, liner instability, technical reasons (explain), and wear.

Failure due to insufficient primary stability most often occurs within the first 2 years. Hence, survival of the cups up to a 2-year follow-up was analyzed. Aseptic loosening due to osteolysis is a late complication that might be influenced by the presence of screw holes. Therefore, we also evaluated the 10-year survival.

### Characteristics of the study population

**Figure 1.**
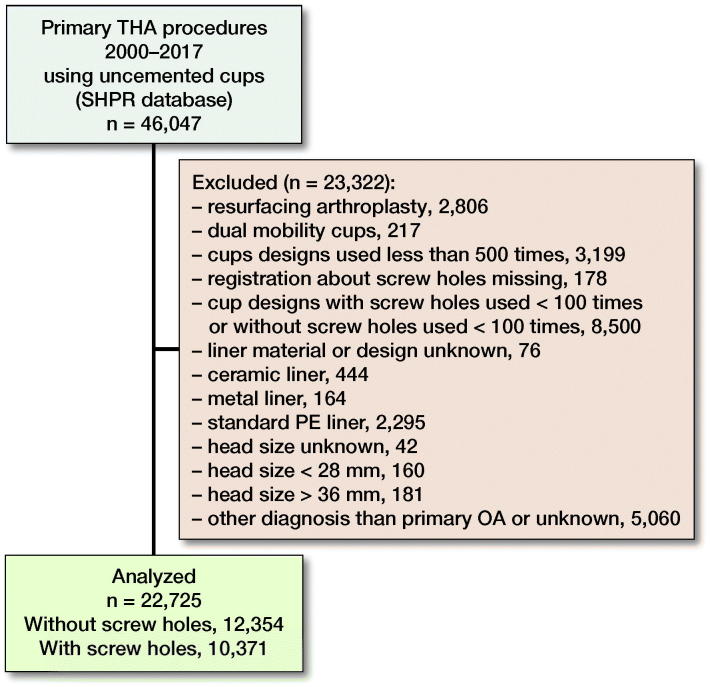
Procedures not included in the final analysis.

**Figure 2.**
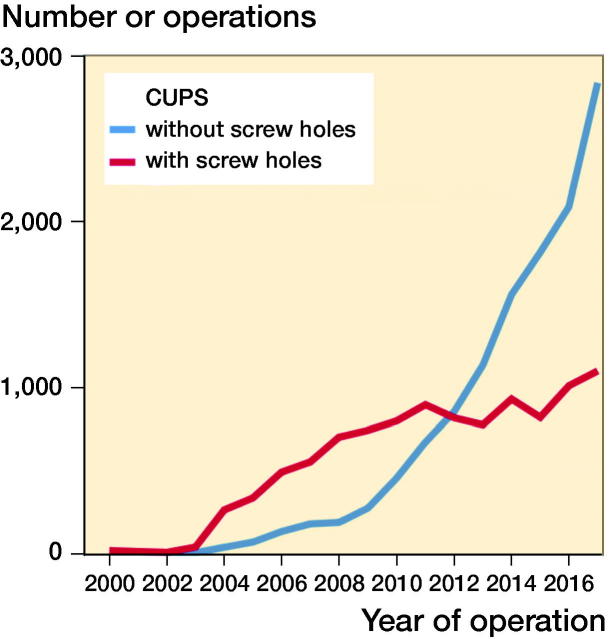
Number of operations per year.

Between January 1, 2000, and December 31, 2017, 46,047 uncemented cups used in primary total hip arthroplasty (THA) were reported to the SHAR, including 63 different cup designs. During the 18-year study period, 30 of these cup designs were reported in less than 100 cases and for 17 cup designs in even less than 10 cases. Only cases using cups that have been frequently used both with and without screw holes and that are still available today were included in this study. After applying the exclusion criteria described in Figure 1, 22,725 hips in 19,840 patients were used for analysis (Table 1), including 8 different cup designs, 12,354 cups without screw holes and 10,371 cups with screw holes (Table 2, see Supplementary data). Both hips were included in 2,885 patients. Cups without screw holes were used in 1,305 of these bilateral THA patients, and cups with screw holes were used in 1,217 bilateral THA patients. The remaining 363 patients had 1 of each type of cup used on each on the 2 sides. The median follow-up time for cups without screw holes was 2.6 years (0–16) and for cups with screw holes was 5.2 years (0–18) (Table 2, see Supplementary data). The number of procedures and the proportion of cups without screw holes increased over time (Figure 2).

**Table 1.** Baseline characteristics of the study population

	Screw holes			
	No n (%)	Yes n (%)	Totals n (%)	p-value a
Sex				
Male	6,916 (56)	5,405 (52)	12,321 (54)	< 0.001
Female	5,438 (44)	4,966 (48)	10,404 (46)	
Age				
< 45	403 (3.3)	406 (3.9)	809 (3.6)	< 0.001
45–64	7,428 (60)	6,395 (62)	13,823 (61)	
65–74	3,662 (30)	2,607 (25)	6,269 (28)	
≥ 75	861 (7.0)	963 (9.3)	1,824 (8.0)	
Side				
Right	6,550 (53)	5,572 (54)	12,122 (53)	0.3
Left	5,804 (47)	4,799 (46)	10,603 (47)	
Approach^b^				
Posterolateral	7,769 (63)	3,252 (31)	11,021 (49)	< 0.001
Direct lateral	4,016 (33)	7,008 (68)	11,024 (49)	
Other	464 (3.8)	97 (0.9)	561 (2.5)	
Type of stem:				
Uncemented	10,580 (86)	8,715 (84)	19,295 (85)	0.001
Cemented	1,774 (14)	1,656 (16)	3,430 (15)	
Cup coating				
No HA	6,662 (54)	3,483 (34)	10,145 (45)	< 0.001
HA	5,692 (46)	6,888 (66)	12,580 (55)	
Head size				
28 mm	1,385 (11)	2,434 (24)	3,819 (17)	< 0.001
32 mm	7,585 (61)	7,128 (69)	14,713 (65)	
36 mm	3,384 (27)	809 (7.8)	4,193 (19)	
Head material				
Metal	9,260 (75)	9,542 (92)	18,802 (83)	< 0.001
Ceramic	3,094 (25)	828 (8.0)	3,922 (17)	
Sum	12,354 (100)	10,371 (100)	22,725 (100)	

Distribution of possible confounding factors between cups with and without screw holes

a Pearson’s chi-square.

b Missing data in 119 cases.

**Table 2.** Number of procedures included per cup design, percentage per group, and median (range) follow-up time in years

	Without screw holes		With screw holes		Total	
Cup design	n (%)	follow-up a	n (%)	follow-up a	n (%)	follow-up a
Regenerex	115 (1)	4.2 (0.1–7.8)	617 (6)	3.3 (0–9.6)	732 (3)	3.6 (0–9.6)
Pinnacle	1,993 (16)	3.1 (0–10.9)	758 (7)	2.2 (0–12.0)	2,751 (12)	2.9 (0–12.0)
Pinnacle Gription	2,916 (24)	1.2 (0–6.3)	448 (4)	0.9 (0–4.8)	3,364 (15)	1.2 (0–6.3)
Trident AD	1,055 (9)	6.3 (0–13.8)	760 (7)	5.3 (0–13.7)	1,815 (8)	6.0 (0–13.8)
Trident hemi	3,092 (25)	2.4 (0–11.1)	213 (2)	2.3 (0–9.8)	3,305 (15)	2.4 (0–11.1)
Tritanium	394 (3)	4.9 (0–8.1)	153 (1)	2.7 (0.1–8.7)	547 (2)	4.2 (0–8.7)
Continuum	2,294 (19)	2.7 (0–7.2)	1,215 (12)	3.1 (0–8.2)	3,509 (15)	2.9 (0–8.2)
Trilogy	495 (4)	7.8 (0–15.6)	6,207 (60)	6.9 (0–17.7)	6,702 (29)	7.0 (0–17.7)
Total	12,354 (100)	2.6 (0–15.6)	10,371 (100)	5.2 (0–17.7)	22,725 (100)	3.4 (0–17.7)

a Median (range) follow-up time in years

There was a significant difference in follow-up time between the groups (p < 0.001, ANOVA).

Operations from 81 different hospitals are included, of which 30 hospitals used cups with screw holes in more than 90% of cases, and 30 hospitals used cups with screw holes in less than 10% of cases.

### Adjusting for confounders

Poor bone quality or abnormal anatomy could lead to the use of cups with screws in primary THA. Neither of these factors is registered in the SHAR. We assumed that older patients and women, in general, have poorer bone quality and patients who had dysplasia or sequelae after hip diseases, as well as patients with other secondary arthrosis, are more likely to have abnormal anatomy. Therefore, we used the patient’s age and sex to adjust the dataset and excluded all diagnoses other than primary osteoarthritis (OA). To various extents, the presence of a hydroxyapatite (HA) coating, the head size, and the head material will contribute to the revision risk (Lazarinis et al. 2010, Cross et al. 2012, Dahl et al. 2012), and these factors were used as covariates in a Cox regression.

Liner material influences the amount of wear. During the last few years, almost exclusively PE liners with x-linked polyethylene (PE) have been used in uncemented cups in Sweden. Hence, only cups with the x-linked PE liner were included in the analysis.

### Statistics

Continuous variables are described as the means, medians, and ranges. Comparisons between groups were performed using independent Student’s t-tests, Welch’s t-tests, or 1-way ANOVA. Categorical data were analyzed with chi-square tests. Survival of the cup was calculated using life tables with 95% confidence intervals (CIs). For comparison of the survival of cups with and without screw holes, the log-rank test was used. Adjusted hazard ratios (HRs) with CIs were calculated using multivariable Cox regression models.

The total observation time comprised 18 years (2000–2017). However, the number of cups at risk for revision after 10 years was only 1,846, and the distribution of cups with and cups without screw holes was unequal between cup design (Table 4, see Supplementary data); therefore, the risk for revision was not calculated beyond 10 years.

**Table 4.** Number of cups at risk of revision in cups with and without screw holes at 2 and 10 years after primary operation

	0 years	2 years	10 years
Without screw holes	12,354	7,228	347
Regenerex	115	85	0
Pinnacle	1,993	1,272	8
Pinnacle Gription	2,916	1,008	0
Trident AD	1,055	904	216
Trident Hemi	3,092	1,758	9
Tritanium	394	330	0
Continuum	2,294	1,430	0
Trilogy	495	441	114
With screw holes	10,371	7,984	1,499
Regenerex	617	450	0
Pinnacle	758	397	34
Pinnacle Gription	448	114	0
Trident AD	760	542	106
Trident Hemi	213	123	0
Tritanium	153	99	0
Continuum	1,215	777	0
Trilogy	6,207	5,482	1,359

The E-value was calculated to define the minimum strength of association of the HR that an unmeasured confounder would need to have with both the treatment and the outcome to fully explain away the association between screw holes and cup revision on the measured covariates (VanderWeele and Ding 2017).

A p-value < 0.05 was considered statistically significant. Statistical analysis was performed using SPSS Statistics software, version 24.0 (IBM Corp, Armonk, NY, USA).

### Ethics, funding, and potential conflicts of interest

The study was approved by the Regional Ethics Committee in Gothenburg (dnr 348-17). No competing interests are declared. The research was funded by SHAR and grants from the regional agreement on medical training and clinical research (ALF) between Västerbotten County Council and Umeå University.

## Results

### Early revisions within 2 years after primary operation

The survival rate with cup revision for aseptic loosening within 2 years was 99.9% (CI 99.8–99.9) for both groups (Table 3). When including all reasons for cup revision, the survival rate for cups without screw holes was 98.6% (CI 98.4–98.8) and for cups with screw holes was 98.4% (CI 98.2–98.7).

**Table 3.** Survival after 2 and 10 years and hazard ratio for revision

Endpoint	2-year survival (95% CI)	10-year survival (95% CI)	Crude HR (0–2 years) (95% CI)	p-value	Adjusted HR (0–2 years) (95% CI)	p-value	Crude HR (0–10 years) (95% CI)	p-value	Adjusted HR (0–10 years) (95% CI)	p-value
Cup revision for aseptic loosening									0.9 (0.5–1.8)	0.8
Without screw holes	99.9 (99.8–99.9)	99.1 (98.6–99.5)	0.8 (0.4–1.7)	0.6	0.6 (0.2–1.8)	0.4	1.2 (0.7–2.1)	0.5	1.0 (ref)	
With screw holes	99.9 (99.8–99.9)	99.5 (99.3–99.7)	1.0 (ref)		1.0 (ref)		1.0 (ref)			
Cup revision for any reason									0.7 (0.5–0.9)	0.004
Without screw holes	98.6 (98.4–98.8)	96.5 (95.8–97.2)	0.8 (0.7–1.0)	0.09	0.6 (0.5–0.8)	0.002	1.0 (0.8–1.2)	0.7	1.0 (ref)	
With screw holes	98.4 (98.2–98.7)	96.8 (96.3–97.2)	1.0 (ref)		1.0 (ref)		1.0 (ref)			
Revision (cup or stem) for any reason									0.7 (0.6–0.8)	< 0.001
Without screw holes	98.0 (97.7–98.2)	95.0 (94.1–95.9)	0.9 (0.7–1.0)	0.09	0.6 (0.5–0.8)	0.001	1.0 (0.8–1.1)	0.6	1.0 (ref)	
With screw holes	97.7 (97.4–98.0)	95.4 (94.9–95.9)	1.0 (ref)		1.0 (ref)		1.0 (ref)			

The adjusted hazard ratio was calculated based on a Cox regression model with gender, age, surgical approach, type of stem fixation, cup coating, head size, head material, and cup design as covariates.

Number of cups at risk for revision in cups without/with screw holes was 12,354/10,371 at 0 years, 7,228/7,984 at 2 years and 3,47/1,499 at 10 years after primary operation.

The crude HR for the risk of cup revision due to aseptic loosening of cups without screw holes compared with cups with screw holes was 0.8 (CI 0.4–1.7). After adjusting for sex, age, surgical approach, type of stem fixation, presence of HA coating, head size, head material, and cup design, we found that screw holes had no influence on cup revision due to aseptic loosening (HR = 0.6, CI 0.2–1.8). The crude HR for cup revision for any reason was 0.8 (CI 0.7–1.0) for cups without screw holes. After adjustment for the covariates, cups without screw holes showed a lower risk for cup revision for any reason with an HR of 0.6 (CI 0.5–0.8) (Table 3) and an E-value of 2.7 (CI 1.8–3.4). The influence of other patient- or prothesis-related factors on the revision rate is presented in Table 5 (see Supplementary data).

**Table 5.** Hazard ratio for cup revision within 2 years for any reason

	No. of hips	No. of revisions	Crude HR (95% CI)	Adjusted HR (95% CI)	p-value
Screw holes:					
Without screw holes	12,354	140	0.8 (0.7–1.0)	0.6 (0.5–0.8)	0.002
With screw holes	10,371	153	1.0 (ref)	1.0 (ref)	
Sex					
Male	12,321	190	1.6 (1.2–2.0)	1.6 (1.2–2.0)	< 0.001
Female	10,404	103	1.0 (ref)	1.0 (ref)	
Age					
< 45	809	13	1.2 (0.7–2.1)	1.1 (0.6–2.0)	0.7
45–64	13,823	186	1.0 (ref)	1.0 (ref)	
65–74	6,269	76	0.9 (0.7–1.2)	1.0 (0.7–1.3)	0.7
> 75	1,824	18	0.8 (0.5–1.2)	0.9 (0.5–1.5)	0.7
Approach					
Posterolateral	11,021	130	1.0 (ref)	1.0 (ref)	
Direct lateral	11,024	159	1.2 (0.9–1.5)	1.0 (0.7–1.3)	0.9
Other	561	3	0.4 (0.1–1.3)	0.5 (0.1–1.5)	0.2
Unknown	119	1	0.6 (0.1–4.6)	0.9 (0.1–6.9)	0.9
Type of stem					
Uncemented	19,295	269	1.0 (ref)	1.0 (ref)	
Cemented	3,430	24	0.5 (0.3–0.8)	0.6 (0.4–1.0)	0.03
Cup coating					
No HA	10,145	158	1.0 (ref)	1.0 (ref)	
HA	12,580	135	0.6 (0.5–0.8)	0.7 (0.5–10.2)	0.2
Head size					
28 mm	3,819	42	1.0 (ref)	1.0 (ref)	
32 mm	14,713	202	1.4 (1.0–1.9)	1.1 (0.8–1.6)	0.6
36 mm	4,193	49	1.2 (0.8–1.8)	1.0 (0.6–1.7)	0.9
Head material					
Metal	18,802	242	1.0 (ref)	1.0 (ref)	
Ceramic	3,922	51	1.1 (0.8–1.5)	1.0 (0.7–1.5)	0.8
Cup design					
Regenerex	732	13	1.6 (0.9–2.8)	1.1 (0.5–2.5)	0.7
Pinnacle	2,751	38	1.3 (0.9–1.9)	1.5 (0.9–2.5)	0.2
Pinnacle Gription	3,364	36	1.2 (0.8–1.7)	1.2 (0.6–2.5)	0.6
Trident AD	1,815	18	0.9 (0.5–1.5)	1.3 (0.7–2.2)	0.4
Trident Hemi	3,305	31	0.9 (0.6–1.4)	1.5 (0.9–2.5)	0.2
Tritanium	547	7	1.1 (0.5–2.4)	1.2 (0.4–3.0)	0.8
Continuum	3,509	72	1.9 (1.4–2.6)	1.8 (1.0–3.3)	0.06
Trilogy	6,702	78	1.0 (ref)	1.0 (ref)	
Hospital					
Frequent user	14,985	169	1.0 (ref)	1.0 (ref)	
Intermediate user	6,482	105	1.4 (1.1–1.7)	1.5 (1.2–1.8)	0.001
Low-volume user	1,258	19	1.3 (0.9–2.0))	1.4 (0.9–2.1)	0.1

The adjusted hazard ratio was calculated based a Cox regression model with the following covariates: screw holes, gender, age, surgical approach, stem fixation, cup coating, head size, head material, and cup design.

In 293 out of 22,725 hips, cup revision, including exchange (or extraction) of the cup, the liner, or both, was performed within the first 2 years after the primary operation. These early revisions were caused by infection in 58% of cases, dislocation in 27%, and aseptic loosening in 9%. Other complications, such as fracture, implant failure, or pain, caused less than 6% of early cup revisions.

The proportion of various reasons for early revision did not differ significantly between cups with and without screw holes (p = 0.2) but differed significantly between cup designs (p = 0.03). For example, all revisions of the Tritanium cup were performed due to infection, while the main reason for revision of the Continuum cup was dislocation (47%).

### Revisions within 10 years after primary operation

The overall 10-year survival rate for aseptic loosening was 99.1% (CI 98.6–99.5) for cups without screw holes and 99.5% (CI 99.3–99.7) for cups with screw holes (Table 3). However, the risk for cup revision due to any reason was still lower for cups without screw holes, with an adjusted HR of 0.7 (CI 0.5–0.9) of E-value of 2.2 (CI 1.5–3.4) (Table 3).

Between the 2- and 10-year follow-up, 152 cup revisions were registered. 41% of these revisions were caused by infection, 26% by dislocation, and 20% because of aseptic loosening. The distribution of these revisions did not differ statistically significantly between patients who underwent unilateral or bilateral operations.

## Discussion

We found similar risk both at 2 years and 10 years for revision because of aseptic loosening between cups with and without screw holes in patients with primary OA. However, the risk for cup revision for any reason at both 2 and 10 years was higher when a cup with screw holes was used.

Screw fixation of uncemented cups increases stability in simulated models (Hsu et al. 2007) and cadaver studies (Won et al. 1995) and is therefore used with the intention to reduce the risk for loosening. This theory might still hold true for patients with abnormal anatomy, fractures, revision settings, and cups without porous coating or trabecular surfaces.

Additional screw fixation of cups with a porous coating or trabecular surfaces has previously been investigated in small populations and did not reduce migration in radiostereometric analysis (RSA) studies (Minten et al. 2016, Otten et al. 2016). A review of 5 articles with a total of more than 1,000 patients and a follow-up time of up to 5 years also did not show any difference in revision rate or osteolysis between cups with and without screw fixation (Ni et al. 2013). Even in the present study with a substantially larger population and operations performed in more than 80 different hospitals, the cups without screw hole did not show a higher risk for early revision due to aseptic loosening. In contrast, we found a higher risk for revision due to any reason for cups with screw holes.

Using screws has some potential risks. Both prolonged operation time (Pepe et al. 2017) and increased likelihood of receiving a blood transfusion (Colacchio et al. 2017) have been reported in recent studies. Inserting screws in the acetabulum might even be a risk for damaging intrapelvic vessels (Ohashi et al. 2017). A report from a smaller group of patients describe a higher risk for osteolysis around cups with screw holes (Iorio et al. 2010).

During the last 2 decades, the number of uncemented cups used per year in Sweden has substantially increased. In the last decade, cups designed without screw holes have been used more often in operations for primary OA than have cups with holes. Data from the current study show that this development did not increase the risk of aseptic loosening. In contrast, we found a higher HR for cup revision for any reason if the cup had screw holes, at both 2 and 10 years. The main reason for both early and late revision was infection. A longer operation time when using screws might have caused a higher risk for infection, and there might also have been differences related to patient selection, which are not possible to adjust for in a register study. The E-value shows that unmeasured confounders need to increase the risk for revision by 2.7 times and must be 2.7 times more common in the group of patients who received a cup with screw hole to fully explain away the differences at 2 years. Of the possible confounding factors available, female sex, the use of cemented stems, and HA coating of the cups reduced the risk for cup revision for any reason. However, these 3 risk-reducing factors were overrepresented in the group of cups with screw holes. Nevertheless, cups with screw holes showed a higher risk for revision due to any reason both at 2 years and 10 years after operation.

The second most common reason for revision in this study was dislocation. The larger the head size, the lower the risk for revision due to dislocation (Hailer et al. 2012). In particular, 22 mm heads, which were used as standard size in the early days of modern hip arthroplasty to reduce wear (Charnley et al. 1969), had a higher risk for dislocation. In cups with cross-linked PE liners, head sizes up to 36 mm do not seem to increase wear (Howie et al. 2016). Therefore, a head size of 28–36 mm, depending on the cup size, seems to be the optimal size when using metal or ceramic heads and a cross-linked PE liner. In this study, only head sizes 28, 32, and 36 mm were included. We did not find any difference in the risk for cup revision for any reason between these 3 head sizes. However, a larger proportion of 28 mm heads was used in the group of cups with screw holes, and this might still have influenced the risk for revision. TMT cups have been reported to have a higher revision rate due to dislocation (Hailer 2018, Laaksonen et al. 2018). Our results are concurrent with these reports.

HA coating has been discussed in several other papers. There is still no consensus on how HA coating influences the risk for cup revision. Several studies have shown an increased revision rate for HA-coated cups (Stilling et al. 2009, Lazarinis et al. 2010). The main reason for the higher revision rate of cups with HA coating seems to be failure of the liner (Lazarinis et al. 2012). Older cup designs have more often been used with HA coating and, at the same time, with standard but not cross-linked PE liners. When adjusting for potential confounders, including the type of liner, in a larger register-based study, a similar risk of aseptic loosening was found for cups with or without HA coating (Lazarinis et al. 2017). Unadjusted data from our study show a slight advantage for cups with HA coating, but when adjusting for several potential confounders, including cup design, there was no statistically significant difference in the risk for revision.

The main limitation of this study is the lack of detailed patient- or surgeon-related information that might have influenced the decision to use screws. Register data do not provide any information regarding the reason why a specific implant was chosen in the individual case. Perhaps surgeons chose cups with the possibility of augmentation with screws in more difficult cases. Excluding all diagnoses other than primary OA reduces the variety of some of these factors. A comparison of the included hospitals showed significant differences in the use of cups with screw holes. It is unlikely that these differences between hospitals can be explained fully by case mix.

Another limitation of our study is that only 2 of the cup designs, with unequal distribution between the groups, had a follow-up time of more than 10 years. Therefore, no reliable analysis was possible beyond 10 years of follow-up, and general conclusions about the long-term consequences of using cups with screw holes should be made with caution. A new analysis of the registry data will be necessary when a sufficient number of cases with several different cup designs have been followed for more than 10–15 years to obtain robust long-term data.

17% of patients were registered with bilateral cups. This subgroup decreases the variance within the groups and can increase the risk for type 1 errors. However, we did not find a statistically significant difference in HR for revision between cups in patients with unilateral THA and cups in patients with bilateral THA.

The incidence of aseptic loosening was very low, making it statistically uncertain to adjust for a large number of potential confounders. The larger number of revisions for any reason gives better statistical strength.

In conclusion, we found that the revision rate for modern and frequently used uncemented cups was very low, and cup revision due to aseptic loosening within 2 years was extremely rare. We could not show that the use of cups designed for additional fixation with screws had any advantages in standard patients. In contrast, cups with screw holes increased the risk of cup revision for any reason. Notably, our study mainly embraces the first decade after the operation. Longer follow-up is needed to evaluate whether this conclusion remains valid during the second decade.

## Supplementary data

Tables 2, 4–5 are available as supplementary data in the online version of this article, http://dx.doi.org/10.1080/17453674. 2019.1599777

VO: study design, data collection, statistical analysis, data analysis, manuscript writing. SM: statistical analysis, data analysis, manuscript writing. KN and SC: data analysis, manuscript writing. JK: study design, data collection, statistical analysis, data analysis, manuscript writing, study supervision.

*Acta* thanks Ross W Crawford for help with peer review of this study.

## Supplementary Material

Supplemental Material

## References

[CIT0001] Aspenberg P, van der Vis H Fluid pressure may cause periprosthetic osteolysis: particles are not the only thing. Acta Orthop 1998; 69(1): 1–4. 10.3109/17453679809002344 9524506

[CIT0002] Charnley J, Kamangar A, Longfield M The optimum size of prosthetic heads in relation to the wear of plastic sockets in total replacement of the hip. Medical Biol Eng 1969; 7(1): 31–9. 10.1007/BF02474667 5771305

[CIT0003] Colacchio N D, Cleary M X, Reid D, Trofa D, Pevear M E, Smith E L J C Direct anterior approach total hip arthroplasty requires less supplemental acetabular screw fixation and fewer blood transfusions than posterior approach. Curr Orthop Pract 2017; 28(4): 404–8.

[CIT0004] Cross M B, Nam D, Mayman D J Ideal femoral head size in total hip arthroplasty balances stability and volumetric wear. HSS J 2012; 8(3): 270–4. 24082871 10.1007/s11420-012-9287-7 PMC3470670

[CIT0005] Dahl J, Soderlund P, Nivbrant B, Nordsletten L, Rohrl S M Less wear with aluminium-oxide heads than cobalt-chrome heads with ultra high molecular weight cemented polyethylene cups: a ten-year follow-up with radiostereometry. Int Orthop 2012; 36(3): 485–90. 21870101 10.1007/s00264-011-1334-3 PMC3291767

[CIT0006] Hailer N 20 years of porous tantalum in primary and revision hip arthroplasty: time for a critical appraisal. Acta Orthop 2018; 89(3): 254–5. 29726759 10.1080/17453674.2018.1463007 PMC6055774

[CIT0007] Hailer N P, Weiss R J, Stark A, Kärrholm J The risk of revision due to dislocation after total hip arthroplasty depends on surgical approach, femoral head size, sex, and primary diagnosis: an analysis of 78,098 operations in the Swedish Hip Arthroplasty Register. Acta Orthop 2012; 83(5): 442–8. 23039167 10.3109/17453674.2012.733919 PMC3488169

[CIT0008] Howie D W, Holubowycz O T, Callary S A The wear rate of highly cross-linked polyethylene in total hip replacement is not increased by large articulations: a randomized controlled trial. J Bone Joint Surg Am 2016; 98(21): 1786–93. 27807110 10.2106/JBJS.15.01248

[CIT0009] Hsu J-T, Chang C-H, Huang H-L, Zobitz M E, Chen W-P, Lai K-A, An K-N The number of screws, bone quality, and friction coefficient affect acetabular cup stability. Med Eng Phys 2007; 29(10): 1089–95. 17194616 10.1016/j.medengphy.2006.11.005

[CIT0010] Iorio R, Puskas B, Healy W L, Tilzey J F, Specht L M, Thompson M S Cementless acetabular fixation with and without screws: analysis of stability and migration. J Arthroplasty 2010; 25(2): 309–13. doi:10.1016/j.arth.2009.01.023. 19303251

[CIT0011] Kärrholm J, Lindahl H, Malchau H, Mohaddes M, Nemes S, Rogmark C, Rolfson O The Swedish Hip Arthroplasty Register Annual Report 2016; 2017.

[CIT0012] Laaksonen I, Lorimer M, Gromov K, Eskelinen A, Rolfson O, Graves S E, Malchau H, Mohaddes M Trabecular metal acetabular components in primary total hip arthroplasty: higher risk for revision compared with other uncemented cup designs in a collaborative register study including 93,709 hips. Acta Orthop 2018; 89(3): 259-64. 29400118 10.1080/17453674.2018.1431445 PMC6055786

[CIT0013] Lazarinis S, Karrholm J, Hailer N P Increased risk of revision of acetabular cups coated with hydroxyapatite. Acta Orthop 2010; 81(1): 53–9. 19968603 10.3109/17453670903413178 PMC2856204

[CIT0014] Lazarinis S, Karrholm J, Hailer N P Effects of hydroxyapatite coating of cups used in hip revision arthroplasty. Acta Orthop 2012; 83(5): 427–35. 22937978 10.3109/17453674.2012.720117 PMC3488167

[CIT0015] Lazarinis S, Mäkelä K T, Eskelinen A, Havelin L, Hallan G, Overgaard S, Pedersen A B, Kärrholm J, Hailer N P Does hydroxyapatite coating of uncemented cups improve long-term survival? An analysis of 28,605 primary total hip arthroplasty procedures from the Nordic Arthroplasty Register Association (NARA). Osteoarthritis Cartilage 2017; 25: 1980–7. 28802851 10.1016/j.joca.2017.08.001

[CIT0016] Minten M J, Heesterbeek P J, Spruit M No effect of additional screw fixation of a cementless, all-polyethylene press-fit socket on migration, wear, and clinical outcome: a 6.5-year randomized radiostereometric analysis follow-up report. Acta Orthop 2016; 87(4): 363–7. 27299418 10.1080/17453674.2016.1190244 PMC4967278

[CIT0017] Ni S H, Guo L, Jiang T L, Zhao J, Zhao Y G Press-fit cementless acetabular fixation with and without screws. Int Orthop 2013. Epub 2013 Aug 28. 10.1007/s00264-013-2075-2 PMC3890122 23982638

[CIT0018] Ohashi H, Kikuchi S, Aota S, Hakozaki M, Konno S Surgical anatomy of the pelvic vasculature, with particular reference to acetabular screw fixation in cementless total hip arthroplasty in Asian population: a cadaveric study. J Orthop Surg (Hong Kong) 2017; 25(1): 2309499016685520. 10.1177/2309499016685520 28498719

[CIT0019] Otten V T, Crnalic S, Röhrl S M, Nivbrant B, Nilsson K G Stability of uncemented cups—long-term effect of screws, pegs and HA coating: a 14-Year RSA follow-up of total hip arthroplasty. J Arthroplasty 2016; 31(1): 156–61. 10.1016/j.arth.2015.07.012 26260783

[CIT0020] Pepe M, Kocadal O, Erener T, Ceritoglu K, Aksahin E, Aktekin C N Acetabular components with or without screws in total hip arthroplasty. World J Orthop 2017; 8(9): 705. 28979854 10.5312/wjo.v8.i9.705 PMC5605356

[CIT0021] Stilling M, Rahbek O, Søballe K Inferior survival of hydroxyapatite versus titanium-coated cups at 15 years. Clin Orthop Relat Res 2009; 467(11): 2872–9. 19330391 10.1007/s11999-009-0796-8 PMC2758968

[CIT0022] VanderWeele T J, Ding P Sensitivity analysis in observational research: introducing the e-value. Ann Intern Med 2017; 167(4): 268–74. 28693043 10.7326/M16-2607

[CIT0023] Won C H, Hearn T, M. Tile Micromotion of cementless hemispherical acetabular components. Does press-fit need adjunctive screw fixation? Bone Joint J 1995; 77(3): 484–9. 7744942

