# Supplementary material for: Uncemented cups with and without screw holes in primary THA: a Swedish Hip Arthroplasty Register study with 22,725 hips
Source: Acta Orthop. 2019 Apr 8;90(3):258–63. doi: 10.1080/17453674.2019.1599777 (PMC6534209; doi:10.1080/17453674.2019.1599777)
Supplement: Supplemental Material [file IORT_A_1599777_SM2104.pdf]

Supplementary data

Table 2. Number of procedures included per cup design, percentage per group, and median (range) follow-up time in years

Cup design	Without screw holes n (%)	Without screw holes follow-up [a]	With screw holes n (%)	With screw holes follow-up [a]	Total n (%)	Total follow-up [a]
Regenerex	115 (1)	4.2 (0.1–7.8)	617 (6)	3.3 (0–9.6)	732 (3)	3.6 (0–9.6)
Pinnacle	1,993 (16)	3.1 (0–10.9)	758 (7)	2.2 (0–12.0)	2,751 (12)	2.9 (0–12.0)
Pinnacle Gription	2,916 (24)	1.2 (0–6.3)	448 (4)	0.9 (0–4.8)	3,364 (15)	1.2 (0–6.3)
Trident AD	1,055 (9)	6.3 (0–13.8)	760 (7)	5.3 (0–13.7)	1,815 (8)	6.0 (0–13.8)
Trident hemi	3,092 (25)	2.4 (0–11.1)	213 (2)	2.3 (0–9.8)	3,305 (15)	2.4 (0–11.1)
Tritanium	394 (3)	4.9 (0–8.1)	153 (1)	2.7 (0.1–8.7)	547 (2)	4.2 (0–8.7)
Continuum	2,294 (19)	2.7 (0–7.2)	1,215 (12)	3.1 (0–8.2)	3,509 (15)	2.9 (0–8.2)
Trilogy	495 (4)	7.8 (0–15.6)	6,207 (60)	6.9 (0–17.7)	6,702 (29)	7.0 (0–17.7)
Total	12,354 (100)	2.6 (0–15.6)	10,371 (100)	5.2 (0–17.7)	22,725 (100)	3.4 (0–17.7)

[a] Median (range) follow-up time in years
There was a significant difference in follow-up time between the groups ($p < 0.001$, ANOVA).

Table 4. Number of cups at risk of revision in cups with and without screw holes at 2 and 10 years after primary operation

	0 years	2 years	10 years
Without screw holes	12,354	7,228	347
Regenerex	115	85	0
Pinnacle	1,993	1,272	8
Pinnacle Gription	2,916	1,008	0
Trident AD	1,055	904	216
Trident Hemi	3,092	1,758	9
Tritanium	394	330	0
Continuum	2,294	1,430	0
Trilogy	495	441	114
With screw holes	10,371	7,984	1,499
Regenerex	617	450	0
Pinnacle	758	397	34
Pinnacle Gription	448	114	0
Trident AD	760	542	106
Trident Hemi	213	123	0
Tritanium	153	99	0
Continuum	1,215	777	0
Trilogy	6,207	5,482	1,359

Table 5. Hazard ratio for cup revision within 2 years for any reason

	No. of hips	No. of revisions	Crude HR (95% CI)	Adjusted HR (95% CI)	p-value
Screw holes:					
Without screw holes	12,354	140	0.8 (0.7–1.0)	0.6 (0.5–0.8)	0.002
With screw holes	10,371	153	1.0 (ref)	1.0 (ref)	
Sex					
Male	12,321	190	1.6 (1.2–2.0)	1.6 (1.2–2.0)	< 0.001
Female	10,404	103	1.0 (ref)	1.0 (ref)	
Age					
< 45	809	13	1.2 (0.7–2.1)	1.1 (0.6–2.0)	0.7
45–64	13,823	186	1.0 (ref)	1.0 (ref)	
65–74	6,269	76	0.9 (0.7–1.2)	1.0 (0.7–1.3)	0.7
> 75	1,824	18	0.8 (0.5–1.2)	0.9 (0.5–1.5)	0.7
Approach					
Posterolateral	11,021	130	1.0 (ref)	1.0 (ref)	
Direct lateral	11,024	159	1.2 (0.9–1.5)	1.0 (0.7–1.3)	0.9
Other	561	3	0.4 (0.1–1.3)	0.5 (0.1–1.5)	0.2
Unknown	119	1	0.6 (0.1–4.6)	0.9 (0.1–6.9)	0.9
Type of stem					
Uncemented	19,295	269	1.0 (ref)	1.0 (ref)	
Cemented	3,430	24	0.5 (0.3–0.8)	0.6 (0.4–1.0)	0.03
Cup coating					
No HA	10,145	158	1.0 (ref)	1.0 (ref)	
HA	12,580	135	0.6 (0.5–0.8)	0.7 (0.5–10.2)	0.2
Head size					
28 mm	3,819	42	1.0 (ref)	1.0 (ref)	
32 mm	14,713	202	1.4 (1.0–1.9)	1.1 (0.8–1.6)	0.6
36 mm	4,193	49	1.2 (0.8–1.8)	1.0 (0.6–1.7)	0.9
Head material					
Metal	18,802	242	1.0 (ref)	1.0 (ref)	
Ceramic	3,922	51	1.1 (0.8–1.5)	1.0 (0.7–1.5)	0.8
Cup design					
Regenerex	732	13	1.6 (0.9–2.8)	1.1 (0.5–2.5)	0.7
Pinnacle	2,751	38	1.3 (0.9–1.9)	1.5 (0.9–2.5)	0.2
Pinnacle Gription	3,364	36	1.2 (0.8–1.7)	1.2 (0.6–2.5)	0.6
Trident AD	1,815	18	0.9 (0.5–1.5)	1.3 (0.7–2.2)	0.4
Trident Hemi	3,305	31	0.9 (0.6–1.4)	1.5 (0.9–2.5)	0.2
Tritanium	547	7	1.1 (0.5–2.4)	1.2 (0.4–3.0)	0.8
Continuum	3,509	72	1.9 (1.4–2.6)	1.8 (1.0–3.3)	0.06
Trilogy	6,702	78	1.0 (ref)	1.0 (ref)	
Hospital					
Frequent user	14,985	169	1.0 (ref)	1.0 (ref)	
Intermediate user	6,482	105	1.4 (1.1–1.7)	1.5 (1.2–1.8)	0.001
Low-volume user	1,258	19	1.3 (0.9–2.0))	1.4 (0.9–2.1)	0.1

The adjusted hazard ratio was calculated based a Cox regression model with the following covariates: screw holes, gender, age, surgical approach, stem fixation, cup coating, head size, head material, and cup design.
